# Outcomes of the California Ban on Pharmaceutical Lindane: Clinical and Ecologic Impacts

**DOI:** 10.1289/ehp.10668

**Published:** 2007-12-11

**Authors:** Elizabeth H. Humphreys, Sarah Janssen, Ann Heil, Patricia Hiatt, Gina Solomon, Mark D. Miller

**Affiliations:** 1 University of California San Francisco–University of California Berkeley Joint Residency Program in General Preventive Medicine and Public Health, Institute for Global Health, San Francisco, California, USA; 2 Health and Environment Program, Natural Resources Defense Council, San Francisco, California, USA; 3 School of Medicine, University of California San Francisco, San Francisco, California, USA; 4 County Sanitation Districts of Los Angeles County, Whittier, California, USA; 5 California Poison Control System, San Francisco Division, Department of Clinical Pharmacy, University of California San Francisco, San Francisco, California, USA; 6 Pediatric Environmental Health Specialty Unit, California Poison Control System-San Francisco, University of California San Francisco, San Francisco, California, USA; 7 Office of Environmental Health Hazard Assessment, California Environmental Protection Agency, Oakland, California, USA

**Keywords:** contamination, head lice, lindane, persistent organic pollutant, pharmaceutical, physician survey, scabies, unintentional ingestions, wastewater

## Abstract

**Introduction:**

There are increasing concerns over the presence and implications of pharmaceutical agents in water. In 2002, California banned pharmaceutical use of lindane because of concerns about water quality, as lindane treatment for head lice and scabies was found to be a significant factor adversely affecting wastewater quality.

**Objectives:**

In this article we describe the effects the ban has had on wastewater quality, unintentional exposures, and clinical practice. This is the first time that a pharmaceutical has been outlawed to protect water quality. As such, this ban provides a rare opportunity to evaluate the possible or potential outcomes of future public health interventions aimed at reducing pharmaceutical water contamination.

**Methods:**

We compiled data on lindane in wastewater treatment plant effluent for several large plants in California and one outside of California. Data on exposures to lindane were obtained from records of the California Poison Control System. We assessed the impact on clinical practice via a survey of 400 pediatricians

**Results:**

Wastewater treatment plant monitoring showed that lindane declined in California after the ban. Similarly, unintentional exposure calls declined. Most physicians were aware of the ban (81%) and had used lindane previously (61%), but they did not notice any difficulties with the ban (78%).

**Conclusions:**

The California experience suggests that elimination of pharmaceutical lindane produced environmental benefits, was associated with a reduction in reported unintentional exposures, and did not adversely affect head lice and scabies treatment. This ban serves as a model for governing bodies considering limits on the use of lindane or other pharmaceuticals.

Approximately 100 different human pharmaceuticals have been identified at low levels in wastewater treatment plant effluents, surface waters, seawaters, groundwater, and some drinking waters from around the world (Fent et al. 2006; Hemminger 2005; Kolpin et al. 2002). Classes of drugs that have been detected include analgesics and anti-inflammatories, beta-blockers, lipid regulators, anti-epileptics, anti-depressants, oral contraceptives, and antibiotics. Intentional flushing of medications down the toilet, rinsing topically applied medications off in the tub or sink, and excretion of medications in urine or feces are the entry points for most pharmaceuticals into wastewater treatment systems. The federal government and a number of states have discouraged disposal of drugs by flushing them down the toilet, but this does not prevent contamination by rinsing or excretion. Although there are no documented health consequences from these exposures, there are concerns about the impact of long-term low-level exposures to medications, especially those that are environmentally persistent, and those that may bio-accumulate in the food chain. Impacts on ecologic systems are also of concern, especially in light of discovery of intersex fish in major waterways in the United States and their association with exposure to endocrine disruptors (Chambers and Leiker 2006). Advanced water treatment technologies can remove many contaminants; however, this technology is expensive and may not be affordable for many municipalities. As communities look for alternative ways to manage pharmaceuticals and persistent chemicals in wastewater, the California ban on lindane deserves scrutiny as a potential approach to improve wastewater quality and limit global contamination with persistent organic pollutants (POPs).

Lindane, the gamma isomer of hexachlorocyclohexane (γ-HCH), is an environmentally persistent organochlorine insecticide manufactured since the 1940s for both agricultural and pharmaceutical purposes. In agriculture, lindane has been used as an insecticide to treat seeds, crops, and lumber/timber, and to treat cattle and other farm animals for ectoparasites. As a prescription medication, lindane is used as a topical treatment for human infestations of head lice and scabies.

Throughout the world, recognition of lindane’s toxicity and its environmental persistence has resulted in an overall decline in use. Lindane has not been produced in the United States for many decades and has undergone progressive limitations on agricultural use [Commission for Environmental Cooperation (CEC) 2006]. In August 2006, the U.S. Environmental Protection Agency (EPA) cancelled all remaining registrations for agricultural uses of lindane (U.S. EPA 2006).

When used as a pharmaceutical, acute exposure to lindane has been reported to cause skin irritation, dizziness, headaches, diarrhea, nausea, vomiting, and, in some instances, convulsions and death (Thomson Micromedex 2006). There have been 3 confirmed deaths and 17 reported deaths associated with lindane use [Food and Drug Administration (FDA) 2003c]. All of the deaths occurred when lindane was used in an off-label manner. Neurologic effects are commonly reported outcomes in the FDA adverse effects database (FDA 2003c). Of the reported neurologic events, 70% included seizure, dizziness, headache, and paresthesias (FDA 2003c). In some instances, lindane has caused seizures after one application given according to package directions (FDA 2003a, 2003b).

Lindane is the least effective common pharmaceutical treatment for head lice when compared *in vitro* with other chemical alternatives including pyrethroids, malathion, or synergized pyrethrins (Meinking et al. 2002). Because of toxicity concerns, in 1995 the FDA advised that lindane be labeled as second line therapy, only to be used after other treatments have failed (FDA 2003c). In 2003, the FDA issued a “black box” public health warning for lindane treatments, reemphasizing that lindane should only be used as second line therapy and recommending use with caution in anyone weighing < 110 lb, the elderly, and those with seizure disorders (FDA 2003c). Despite the cancellation for agricultural use, demonstrated toxicity in humans, and low efficacy in treating pediculosis (Meinking et al. 2002), lindane continues to be available by prescription in the United States.

Lindane is a known contaminant in waste-water. Because head lice and scabies treatments are rinsed down the drain after use, lindane readily enters wastewater treatment plants. Wastewater treatment plants are not designed to remove lindane; therefore, much of the lindane passes through and enters downstream lakes, rivers, and the ocean (U.S. EPA 2002). California has stringent water quality standards for lindane, including a criteria of 19 ppt for existing or potential drinking water sources (U.S. EPA 2000). This standard is based on long-term human cancer risk from ingestion. Wastewater treatment engineers in Los Angeles, California, calculated that a single treatment for head lice or scabies contains enough lindane to bring 6 million gallons of water above this California water quality standard [County Sanitation Districts of Los Angeles County (CSDLAC) 2001]. To address these concerns, the CSDLAC, the City of Los Angeles, and the National Pediculosis Association jointly conducted an outreach campaign in 1999 to provide information to clinicians on alternatives to lindane and to recommend limiting its use (CSDLAC 2000). The outreach campaign consisted of three direct mailings to target audiences and mass-media exposure. Direct mailings included flyers (in seven languages), refrigerator magnets, and head lice combs. Presentations were made addressing the issues of the campaign. Mass media included newspaper, radio, and television coverage. The target audiences included doctors, hospitals, pharmacists, school nurses, and day care centers in Long Beach and Burbank, California. A relevant website and a toll-free hotline were also established.

In 2000, the California legislature passed, with no opposition on record, a ban on the sale of all pharmaceutical lindane products effective 1 January 2002 (State of California 2000). To determine the potential impact of this ban on water quality, we obtained data from wastewater treatment plants on waste-water lindane concentrations. Also, to determine the number of acute poisonings due to lindane before and after the ban, we reviewed telephone calls to the California Poison Control System. To more systematically investigate the impact of the lindane ban on prescribing practices of health care providers’ in California, we surveyed pediatricians 3 years after the ban’s effective date.

## Methods

### Wastewater concentrations

We examined annual mean concentrations of lindane (in parts per trillion) for several large treatment plants in California. Historical water-lindane concentrations were obtained directly from the following California agencies: CSDLAC’s Joint Water Pollution Control Plant, serving > 3 million people in Los Angeles County; the City of Los Angeles’ Hyperion Treatment Plant, also serving > 3 million people in Los Angeles County; the Plants 1 and 2 of the Orange County Sanitation Districts, serving 2.5 million people in Orange County; and the San Jose/Santa Clara Water Pollution Control Plant, serving 1.5 million people in Santa Clara County. Concentrations of lindane entering the plants were examined except for the San Jose/Santa Clara Water Pollution Control Plant, where adequate data were not available. For that treatment plant, we examined concentrations of lindane exiting the plant. Because lindane is not widely analyzed at wastewater treatment plants outside of California using sensitive analytical methods, limited wastewater lindane data is available outside of California for comparison. However, we obtained data for the Clermont County Sewer District’s Middle East Fork Wastewater Treatment Plant, serving approximately 30,000 people in Clermont County, Ohio. Routine monitoring of wastewater for lindane at these treatment plants was performed monthly or quarterly, and was based on samples taken over a 24-hr period.

### California Poison Control System calls and prescribing trends for lindane

We searched the California Poison Control System case management database using Visual Dotlab, version 4.3.1 (WBM Software, Fresno, CA) for years 1998–2006 for calls related to unintentional exposures to lindane. We used product-specific codes from Poisindex (Thomson Healthcare 2007) for personal care products containing lindane to identify 21 shampoos, creams, and lotions. A tally of annual calls related to all unintentional exposures was obtained as a denominator.

To examine lindane prescribing trends in California, Medi-Cal fee-for-service pharmacy paid claims data for lindane were compiled for the fiscal years 1997–2002 (California Department of Health Services 2007). Nationwide data on the total number of lindane prescriptions by calendar years 1997–2006 were also compiled (Verispan Inc. 2007).

### Survey

We developed a written survey to elicit information about characteristics of provider practices, provider awareness and perception of the California lindane ban, and current treatment preferences for head lice and scabies. We obtained approval from the Committee on Human Research at the University of California, San Francisco, prior to mailing the survey. Among a population of 4,179 non-emeritus members of the American Academy of Pediatrics, California district, 400 members were selected at random to receive the survey. Each selected participant was mailed three separate surveys with a return envelope 1 month apart. We analyzed the data using Stata, version 9 (StataCorp, College Station, TX).

## Results

### Wastewater concentrations

**Figure 1:**
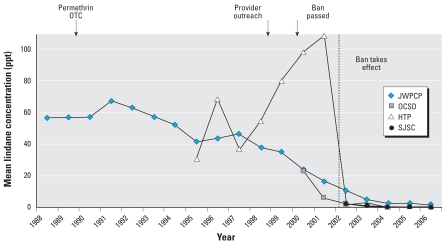
Mean lindane concentrations at four California wastewater treatment plants: Joint Water Pollution Control Plant (JWPCP), Hyperion Treatment Plant (HTP), Orange County Sanitation Districts’ Plants 1 and 2 (OCSD), and San Jose/Santa Clara Water Pollution Control Plant (SJSC). All data are for influent except for SJSC, which are for effluent. The California standard for lindane in surface water bodies that are existing or potential drinking water sources is 19 ppt. Arrows indicate when permethrin became OTC (1990), the outreach campaign in Los Angeles County began (1999), and the ban was passed (2000); the ban took affect in 2002 (dashed line).

Before Los Angeles County outreach efforts on pharmaceutical lindane began in 1999, the average wastewater concentration of lindane was 36 ppt. Although the concentration has declined steadily since that time, it remained elevated at several major California wastewater treatment plants at the time the lindane ban in California was enacted. By 2006, 4 years after the ban took effect, lindane concentrations had dropped to almost undetectable concentrations in California. There is limited availability of lindane wastewater data outside of California, but data from one Ohio treatment plant (Clermont County Sewer District’s Middle East Fork Wastewater Treatment Plant, Batavia, OH) indicates that lindane concentrations remained significantly elevated in Ohio after the California ban was enacted. Figure 1 shows the mean concentration of lindane at the California treatment plants.

### California Poison Control System exposure calls and prescribing trends for lindane

**Figure 2:**
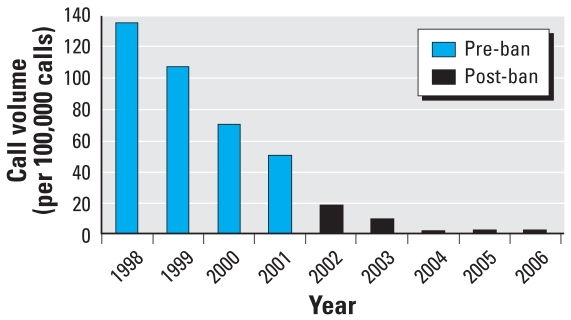
Annual number of calls regarding unintentional exposures to lindane per 100,000 calls, 1998–2006. Data from the California Poison Control System.

In 1998, there were 135 calls reporting unintentional lindane exposure per 100,000 calls to the California Poison Control System. This volume declined somewhat in 2001 (50 calls/100,000) and then fell to near zero in the years following the ban (2 calls/100,000 per year for 2004–2006). Figure 2 shows annual calls to the California Poison Control System for 1998–2006 for unintentional exposures related to lindane.

**Figure 3:**
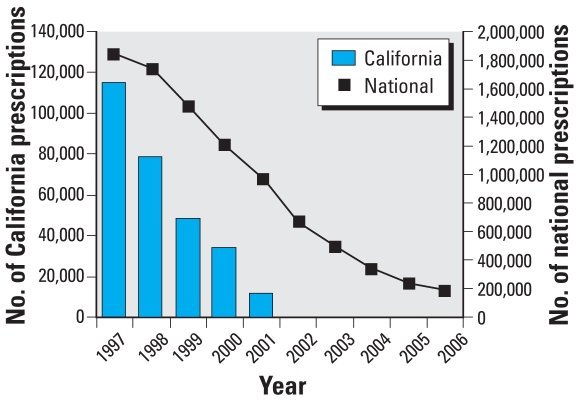
Number of prescriptions of lindane according to Medi-Cal data for 1997–2002 compared with nationwide prescriptions for years 1997–2006.

Prescriptions for lindane filled by the Medi-Cal state insurance program dropped from > 114,000 in 1997 to 11,366 in 2001 and 34 in 2002 (reflecting delayed payment for pre-ban prescriptions) paralleling the decline in wastewater concentrations (Figure 3). Nationwide prescriptions for lindane during the same period declined similarly. In January 2002 California sales ended abruptly, coinciding with the ban. In contrast, since 2002 the rate of decline in national sales has slowed, as illustrated in Figure 3.

### Surveys

Of the 400 mailed surveys, 171 (43%) were returned after three mailings. No information was available for the non-responders. Thirty-two surveys from non-practicing physicians were excluded from the final analysis. In addition, 4 incomplete surveys were dropped from the group. The analysis was performed on the remaining 135 responses.

#### Responder characteristics

Table 1 describes the practice characteristics of the respondents. The majority (77%) of responding health providers practiced > 30 hr/week. Over one-half (55%) of the pediatricians practiced in a group private practice, 14% were in a health maintenance organization setting, and 13% of respondents were in solo practice. Approximately one-half (53%) of respondents were in practice for < 15 years.

Pediatricians differed substantially in the number of cases of head lice and scabies they typically manage in their practice. For head lice, 70 respondents (52%) reported managing 3–14 cases in the last 3 months, and 60 respondents (45%) managed < 2 cases; only 3% of the providers managed > 15 cases of head lice in the last 3 months. Similarly, for scabies, 50 pediatricians (37%) managed between 3 and 14 cases of in the last 3 months, and 79 (59%) managed ≤ 2 cases; only 4% managed > 15 cases of scabies in the last 3 months.

#### Response to lindane ban

More than one-half (61%) of pediatricians reported using lindane before the ban, and the vast majority (81%) were aware of the ban. Of the providers who reported using lindane prior to the ban, virtually all (94%) reported changing their prescribing practices as a result of the ban.

Most respondents (78%) did not notice any difficulties after the lindane ban. However, 30 providers did report difficulties after the ban. Of these, most used lindane before the ban (26 of 30; 87%) and only 4 of 30 did not report prior lindane use. Those providers who reported that they had used lindane pre-ban and noticed difficulties after the ban were far more likely to be in solo private practice (35% vs. 7%) and to have been in practice > 15 years (58% vs. 44%). Providers reporting difficulty after the ban cited resistant lice as the main reason (97%); however, overall reports of resistant scabies were minimal (5%), as were increased cases of lice (7%) or scabies (1%). There were no significant differences among volume of head lice or scabies cases seen in the previous 3 months between providers who reported difficulties and those who did not.

#### Treatment preferences

The majority of respondents (69%) stated their first-line treatment preference for head lice was 1% permethrin, followed by 5% permethrin (9%) and other over-the-counter (OTC) methods including pyrethrum (8%). Respondent preference for second-line head lice treatment was malathion (51%), followed by 5% permethrin (19%). For scabies treatment, the majority (92%) of respondents expressed preference for 5% permethrin (92%), followed by crotamiton (5%). Second-line treatment preference for scabies included crotamiton (32%), followed by 5% permethrin (25%), malathion (21%), and others (22%).

## Discussion

### Wastewater concentrations

Because there was little to no agricultural use of lindane in urban areas of California, elevated wastewater concentrations of lindane were attributed to pharmaceutical lindane usage (CSDLAC 2001).

As Figure 1 illustrates, average concentrations of lindane were declining after 1991, paralleling reductions in prescriptions filled in California by Medi-Cal (Figure 3) and likely reflecting the availability of effective and safe OTC alternatives. Permethrin, for instance, was first available in 1986 and made OTC status in 1990. Although we were unable to establish historical retail prices of lindane, the lowest average wholesale price (AWP) of lindane shampoo relative to that of permethrin went from 20% to 110% from 1990 to 1999 (Kapusnik-Uner J, personal communication). In addition to the introduction of alternative treatments and public health advisories, medical treatment recommendations in the literature and price increases of lindane may have also contributed to the gradual decline in use of lindane.

After the pharmaceutical lindane ban went into effect, lindane concentrations at California wastewater treatment plants dropped to essentially nondetectable levels. Although there is limited data available outside of California for comparison, one Ohio wastewater treatment plant demonstrated significantly elevated lindane concentrations after the California ban was enacted. This suggests that the ban played a major role in the decreased California waste-water concentrations relative to other factors, such as the cost of pharmaceutical lindane and availability of alternatives.

### Unintentional ingestion and prescribing trends for lindane

Lice and scabies infestations are a worldwide problem, and are especially prevalent in institutions such as schools, prisons, and nursing homes. Infestations are usually not life-threatening, but they can be persistent and recurring, and they can cause considerable frustration and embarrassment in families. Whereas lindane was once an inexpensive and effective treatment, it is now more expensive than many alternatives (West 2004) and has been associated with widespread resistance throughout the world (Heukelbach and Feldmeier 2006; Ko and Elston 2004).

A recent report shows that prescriptions for lindane in the United States have declined by 87% over the last 12 years (U.S. EPA 2007). Yet, there were 242,000 prescriptions written for lindane in 2005 (U.S. EPA 2007) and > 186,000 in 2006 (Verispan Inc. 2007), and 870 unintentional ingestions of lindane occurred in the United States during the 5-year period 1998–2003 [Centers for Disease Control and Prevention (CDC) 2005b]. Although there was a dramatic decline in lindane prescriptions filled under the Medi-Cal program in California in the 5 years prior to the ban, there were still > 11,000 filled in the year before the ban. Despite lindane’s use as a second-line drug, unintentional ingestions from lindane were more likely to produce illness than ingestions of all alternative medications combined (pyrethrin/piperonyl butoxide, permethrin, and malathion) (CDC 2005b). In contrast, calls related to lindane exposure to the California Poison Control System declined gradually from 1998 to 2002, but went to near zero after the ban. This information highlights the fact that, although the pharmaceutical use of lindane in states other than California has declined, there is still a significant volume of use and continued morbidity from unintentional exposures.

### Summary of survey results

Three years after pharmaceutical use of the pesticide lindane was banned in California, our survey of practicing California pediatricians indicated that > 80% of physician respondents were aware of the ban, and a similar majority reported no difficulties complying with the ban. Despite outreach efforts by the State Department of Health Services and county public health officials, nearly two-thirds of pediatricians were prescribing lindane at least occasionally before the ban and had to change their prescribing practices as a result of the ban. The minority of providers who were using lindane and noticed difficulties after the ban were more likely to be in solo practice and to have been in practice > 15 years, suggesting a subpopulation of pediatricians who may benefit from education about alternative treatments for head lice and scabies. There was concordance among providers for current first line treatments for head lice and scabies.

Most providers did not report an increase in resistance of lice or scabies following the ban on lindane. One limitation of our survey is that it was not sensitive enough to distinguish between an increase in resistance predating the ban from any additional resistance temporally associated with the ban or thought to be related to the ban. Most chemical treatments for pediculosis will result in resistance over time (Downs 2004). Written comments on our survey from providers suggest that either there was no additional increase in resistance after the ban or that any increase was unrelated. For instance, written responses from providers included these comments: “there seems to be an inexplicable decrease in both infestations”; “I think resistance and ban of Kwell are entirely unrelated”; and “only seeing rare clinical challenges now.” As well, the California Department of Public Health has not identified an impact of the ban on either head lice or scabies outbreaks (Husted S, personal communication).

This study is, to our knowledge, the first evaluation of the clinical and environmental effects of the California lindane ban. The main limitation of our survey was a survey response < 50%, although this response rate is similar to that from other published studies using mailed surveys (Asch et al. 1997; McMahon et al. 2003). There is no information about the nonresponders. This low response rate could introduce bias. For example, if providers who experienced problems were more likely to respond to the survey, this would have overestimated reported difficulties following the lindane ban.

### Alternatives to lindane for head lice/scabies treatment

The current recommended first-line treatment for head lice is OTC 1% permethrin (Frankowski and Weiner 2002). Pediatricians in the California survey generally seemed to be aware of this and adhere to the guidelines. A recent Cochrane Review (Dodd 2001) found no evidence that any one pediculocide, including malathion, permethrin, and synergized pyrethrins, was more effective than another, although only 4 of 71 randomized, placebocontrolled studies met the inclusion criteria. Oral ivermectin has also been used when topical treatments cannot be used or when all other therapies have failed, although it is currently not FDA approved for this use (The Medical Letter 2005).

A complete review of alternatives for the treatment of head lice and scabies is beyond the scope of this article; however, several recent publications provide such a discussion (Jones and English 2003; Karthikeyan 2005; Meinking 2004; Walker and Johnstone 2000). Several recent uncontrolled studies on nonchemical treatments for head lice—relying on suffocation and desiccation—also show promise (Goates et al. 2006; The Medical Letter 2005; Pearlman 2004). In another recent small single-blinded, randomized study comparing common pediculocides to wet combing (nit removal by using a fine-toothed comb through wet hair), Hill et al. (2005) found wet combing to be effective. These methods are preferable because they are not toxic to humans or the environment and are not susceptible to the development of resistance.

### Environmental concerns

Currently, lindane may only be sold in the United States for use as a second-line treatment for head lice and scabies. However, the continued use and production of lindane raises international environmental pollution concerns and ethical issues.

For every ton of lindane that is produced, approximately 9 tons of toxic waste by-products are generated (CEC 2006). Lindane is the γ-isomer of HCH and is isolated from a mixture of eight isomers in technical-grade HCH (CEC 2006). None of the other HCH isomers are used commercially, and several are significantly more toxic and persistent than lindane itself, creating a disposal problem that has been poorly managed in many countries. Lindane production and use has resulted in contamination of products significant to children, such as butter and milk (Pardio et al. 2003; Waliszewski et al. 2003). In both animal and human studies, lindane and other HCH isomers have been associated with toxic health effects, including neurotoxicity, increased cancer risk, reproductive harm, and immune suppression (Agency for Toxic Substances and Disease Registry 2005; International Agency for Research on Cancer 1998). Although long lived in the environment, studies have shown that in countries that have restricted or banned lindane, levels of HCH in breast milk have declined over time (Jensen and Slorach 1991; Konishi et al. 2001; Schade and Heinzow 1998). Similarly, biomonitoring data from the United States found levels of lindane below the limits of detection, and lower than in people from many other countries (CDC 2005a). The β-HCH isomers are still found in measurable concentrations in Americans and were higher in Mexican Americans (CDC 2005a).

Over the past two decades, there has been a steady decline in the production and use of lindane. Worldwide production of lindane is estimated to have decreased from 38,000 tons/year in 1986 to approximately 3,222 tons/year during 1990–1995 (International POPs Elimination Network 2007). More recent figures are not available. However, it has been estimated that between 2 and 4.8 million tons of HCH waste by-products are present worldwide (Vijgen 2006). These waste products are highly persistent chlorinated compounds; thus, there is no easy and effective way to dispose of them or remediate sites of production, creating a costly and hazardous situation. Production of lindane has moved from industrialized to developing countries, which raises ethical issues because the manufacturing country becomes the dumping ground for the waste. Documentation about production is sparse. Because it is joining the European Union, Romania is slated to discontinue production at the end of 2007. The remaining lindane production sites are thought to be only India and China (CEC 2006; Schade and Heinzow 1998).

Lindane is registered for use in 17 countries, has been completely banned in > 50 countries, and has restricted use in 33 countries (CEC 2006). In recognition of the global pollution resulting from POPs such as lindane, there have been international efforts to regulate and eliminate these substances. Mexico, the United States, and Canada, for instance, have collaborated in the North American Regional Plan to eliminate or ban the use of lindane where warranted and reduce the risks from exposure to HCH isomers (CEC 2006). In addition, Mexico has nominated lindane and other HCH isomers as candidates for the Stockholm Convention, a global treaty to protect human health and the environment from POPs.

In summary, there are safer and more effective treatment alternatives for head lice and scabies. The experience in California has resulted in ecologic benefits, including the virtual elimination of lindane from California wastewater, and in the reduction of unintentional exposure calls to the Poison Control System. Our survey results suggest that the ban on the pharmaceutical use of lindane has not posed a significant problem for clinicians. Use and wastewater contamination did decrease in California during the years before the ban, likely resulting from California public outreach efforts, the FDA advisories, recommendations in the medical literature, and the availability of alternatives. However, use continued; it was ultimately the legislative ban that was correlated with improvements in California waste-water quality, a decrease in unintentional exposure calls, and the cessation of clinician use of lindane. Given the recognition of lindane and other HCH isomers as toxic and persistent chemicals with health consequences, coupled with the ethical issues of manufacturing in developing countries for use elsewhere, the harms of use and production may outweigh any residual benefit from maintaining it as a second-line therapy.

## Figures and Tables

**Table 1:** Pediatrician survey respondent characteristics.

	No. (%)
Practice type	
Solo	17 (13)
Group or private	74 (55)
Health maintenance organization	19 (14)
Federal, military, or public	7 (5)
Academic	11 (8)
Other	7 (5)
Hours practiced per week	
≥ 30	104 (77)
< 30	31 (23)
Years in practice	
≥ 15	63 (47)
< 15	72 (53)

## Appendix 1

Information on lindane pricing is based on available average wholesale prices (AWP) from 1990 to 1999.

AWP [is the] average wholesale price or wholesaler’s published price to buying entities (pharmacies, hospitals, etc. and may vary by reporting source); AWP represents published catalogue or list prices and may not represent actual transactional prices.

Reprinted with permission by First DataBank, Inc. All Rights Reserved 2007. Pricing methodology available online at: http://www.firstdatabank.com/support/rcs/policies/pricing/.
